# Supplementary material for: A Comprehensive Assessment of Ultraviolet-Radiation-Induced Mutations in Flammulina filiformis Using Whole-Genome Resequencing
Source: J Fungi (Basel). 2024 Mar 20;10(3):228. doi: 10.3390/jof10030228 (PMC10971301; doi:10.3390/jof10030228)
Supplement: Supplementary file 1 [file jof-10-00228-s001.zip › Supplementary Material S8/KEGG annotation/out/64381550635650.os/KO/out_map/map05410.html]

KEGG PATHWAY: Hypertrophic cardiomyopathy (HCM) - Reference pathway


|  |  |
| --- | --- |
| **Hypertrophic cardiomyopathy (HCM) - Reference pathway** |  |

[
Pathway menu
| Organism menu
| Pathway entry
| Show description
| User data mapping
]

Hypertrophic cardiomyopathy (HCM) is a primary myocardial disorder with an autosomal dominant pattern of inheritance that is characterized by hypertrophy of the left ventricles with histological features of myocyte hypertrophy, myfibrillar disarray, and interstitial fibrosis. HCM is one of the most common inherited cardiac disorders, with a prevalence in young adults of 1 in 500. Hundreds of mutations in the genes that encode protein constituents of the sarcomere have been identified in HCM. These mutations increase the Ca2+ sensitivity of cardiac myofilaments. Increased myofilament Ca2+ sensitivity is expected to increase the ATP utilization by actomyosin at submaximal Ca2+ concentrations, which might cause an imbalance in energy supply and demand in the heart under severe stress. The inefficient use of ATP suggests that an inability to maintain normal ATP levels could be the central abnormality. This theory might be supported by the discovery of the role of a mutant PRKAG2 gene in HCM, which in active form acts as a central sensing mechanism protecting cells from depletion of ATP supplies. The increase in the myfilament Ca2+ sensitivity well account for the diastolic dysfunction of model animals as well as human patients of HCM. It has been widely proposed that left ventricular hypertrophy is not a primary manifestation but develops as compensatory response to sarcomere dysfunction.

|  |  |
| --- | --- |
| Reference pathway | 100% |
